# Hybrid versus distance learning environment for a paediatric dentistry course and its influence on students’ satisfaction: a cross-sectional study

**DOI:** 10.1186/s12909-022-03417-4

**Published:** 2022-05-05

**Authors:** Yasmin Mohamed Yousry, Maha Moussa Azab

**Affiliations:** 1 Department of Pediatric Dentistry, Faculty of Dentistry, Cairo University, Cairo, Egypt; 2 Department of Pediatric Dentistry, Faculty of Dentistry, Fayoum University, Fayoum, Egypt; 3 Discipline of Pediatric Dentistry, School of Dentistry, Newgiza University NGU, Giza, Egypt

**Keywords:** Dental education, Distance learning, Satisfaction, Paediatric dentistry

## Abstract

**Background:**

During the novel COVID-19 pandemic, many universities adopted distance and hybrid learning as a modification to their teaching methods to ensure continuity of education, abiding by the worldwide recommendations of social distancing.

**Aim:**

To compare learning environments created through hybrid learning versus distance learning, to deliver paediatric dentistry course, and to assess the correlation between the created learning environment and students’ satisfaction.

**Method:**

In this cross-sectional study, students enrolled in a hybrid paediatric dentistry course were asked to participate in an electronic survey. The learning environment was assessed using Distance Educational Learning Environment Survey (DELES), students’ satisfaction was assessed using Satisfaction Scale (SS). Retrospective data for distance learning course was used for comparison. Ordinal data were compared using Mann-Whitney U test. Spearman’s rank order correlation coefficient was used to correlate students’ satisfaction with DELES. Multiple regression analysis was used to predict satisfaction.

**Results:**

A total of 376 students’ data were considered in the study. Hybrid learning had significantly higher scores than distance learning in 3 DELES scales. There was a statistically significant weak positive correlation between satisfaction and DELES. Multiple regression analysis model was statistically significant and accounted for (22.8%) of the variance in students’ satisfaction. Only “Instructor support” (*p* = 0.001) and “Student autonomy” (*p* < 0.001) had a significant effect on satisfaction.

**Conclusion:**

This study supports the superiority of a hybrid learning environment over a complete distance learning environment, it also shows that satisfaction is correlated and can be predicted by the created learning environment.

**Trial registration:**

This study has been registered on clinicaltrials.gov on 21 May 2020 with an identifier: NCT04401371.

**Supplementary Information:**

The online version contains supplementary material available at 10.1186/s12909-022-03417-4.

## Background

The worldwide outbreak of the novel COVID-19 virus has forced countries to take necessary measures to tackle the spread of the disease. Earlier with the appearance of the disease, due to safety concerns and the need for ongoing social distancing, most governments around the world have suspended on-campus education, leading to significant disruption to the provision of dental education.

During the academic year 2019 /2020, the faculty of dentistry, Cairo university had made appropriate and timely modifications to their teaching methods to ensure continuity of education during the pandemic. The decision made to cope with this situation was to continue with lectures and group sections, using distance learning abiding by the worldwide recommendations of limiting clinical dental practice due to the high risk of exposure to coronavirus through aerosol-generating procedures [1]. However, in the following academic year 2020/2021, social distancing guidelines started to relax world wild, allowing the implementation of hybrid learning. The appearance of newly different teaching methods resulted in an abrupt change in the learning environment, that might affect students’ ability to learn.

The term “learning environment” is a complex term that can be defined broadly as “factors in which students’ learning processes are embedded.” It refers to the variety of physical settings, contexts, and cultures in which students learn. The goal is to create a total learning environment that maximizes students’ learning abilities [2].

There is no single ideal learning environment, students can learn in a variety of ways, in a variety of contexts. Creating a learning environment for students in a specific course or program is probably the most creative aspect of teaching. However, from our constructivist’s point of view, creating an appropriate learning environment from the perspective of an instructor only is not enough. Students should participate in and affect their learning process; it is very important to consider learning environments from the students’ perspectives.

Distance learning has shown many advantages. It is a flexible educational option that allows a student to stay connected even during long absences, a student can schedule his attending time according to his convenience, determine his pace of work, and make decisions involving both planning and implementation [3].

However, an interactive social environment where learner-learner, learner-instructor, and learner-content interactions are organized in an educational setting that promotes and facilitates learning; is considered a challenge in pure distance learning due to the physical isolation of both instructors and students [4].

Hybrid learning is an educational method that combines on-campus and remote online learning at the same time. In our experience, some parts of the curriculum were taught online, in the form of recorded or interactive sessions; for other parts, students were obliged to attend on-campus. Hybrid learning promotes flexibility while also providing the social aspect of education. It has been reported that students usually place a higher value on interaction with instructors and peers in face-to-face sessions and on interaction with content in online sessions [5].

Interaction has been considered one of the most critical challenges in both traditional education and distance learning. Several studies have shown the positive influence of social environments on encouraging students’ interaction and satisfaction with distance courses [6].

Satisfaction has been identified as a critical measure to the quality and ongoing success of educational courses, it was reported that students with higher levels of satisfaction towards different aspects of distant learning show lower attrition rates, higher persistence in learning, higher motivation, and significantly higher levels of knowledge than others with lower level of satisfaction [7].

Limited studies were carried out to assess the distance learning experiences in the field of dentistry; Ramlogan et al. [8], compared knowledge and skills attained in some clinical periodontology exercises when delivered through videos versus live lectures, students had preferred integration of videos in the process rather than considering it as a substitution for conventional lectures. Asiry [9] found positive attitudes from students regarding distance learning for a preclinical orthodontic course, where students mostly preferred a combination of distance learning and traditional learning.

The educational environment is mainly intended for students and should accommodate their needs. Having insight into students’ perspectives of the created environments is very important to identify those components that need to be considered in teaching the course in the future; aiming to optimize the student’s ability and satisfaction with learning. Course coordinators must use learners’ feedback on courses to be able to co-ordinate and modify the most advantageous learning experiences which will result in an improvement of the effectiveness of education; creating an appropriate learning environment for paediatric dentistry course during the COVID-19 crisis is considered a relatively new experience, so this study aims to, 1) Compare learning environments created through hybrid learning versus distance learning, to deliver paediatric dentistry course, 2) Assess the correlation between created learning environment and students’ satisfaction.

## Materials and methods

### Study setting and eligibility criteria

The target population in this study was senior dental students (final-year students) enrolled in paediatric dentistry course at Cairo University.

#### Inclusion criteria

The participant should be a final-year dental student.

Students who have received and completed paediatric dentistry course.

#### Exclusion criteria

Students who were unwilling to participate and refused to fill out an informed consent.

Students with missing scores, who didn’t answer all the survey questions.

### Sample size

This study is a census study, all students enrolled in paediatric dentistry hybrid course during the academic year 2020 /2021 (*n* = 270), were asked to participate in this survey. One of the most significant advantages of a census survey is that all students have an opportunity to participate, making results obtained from a census study more accurate and reliable [10].

### Instrument

The tool chosen to collect data about the learning environment created in paediatric dentistry hybrid course was Distance Educational Learning Environment Survey (DELES) [11]. Satisfaction Scale (SS) was used to assess students’ satisfaction with the hybrid course [12].

DELES is a Likert-type survey, DELES scales were developed, considering previous tools and experts’ opinions regarding the essentials for a successful distance education learning environment. This was followed by adapting items used in previous tools and creating new items for the preidentified scales [11]. The process was reviewed and validated by a panel of experts followed by field testing and factor analysis, resulting in the end form of DELES consisting of six scales, “Instructor support”: 8 items, “Student interaction and collaboration”: 6 items, “Personal relevance”: 7 items, “Authentic learning”: 5 items, “Active learning”: 3 items, and “Student autonomy”: 5 items; building up a 34-item survey, with responses: 5 = always, 4 = often, 3 = sometimes, 2 = seldom, 1 = never. SS is an eight-item scale with responses: 5 = strongly agree, 4 = agree, 3 = neither agree nor disagree, 2 = disagree, 1 = strongly disagree. Scores were calculated by adding the answers of all items of a scale.

The survey used in the current study consisted of two closed-type questions to determine gender and age. Then the following sections included all DELES scales except (personal relevance). Finally, the last section consisted of SS.

The English versions of DELES and SS were used to create the electronic edition of the questionnaire using the Google forms application (https://docs.google.com/forms/u/0/).

At the end of paediatric dentistry hybrid course (May 2021), students were informed through e-mail about the full details and purpose of the study and that their participation in the study will be voluntary and anonymous; additionally, they were informed that they will be granted full access to published data.

Two days later a link to the questionnaire was e-mailed to all students through their official e-mails, together with a confidential and voluntary consent form. Finally, after 3 days a reminder e-mail was sent thanking those who had already responded and asking students who has not already participated to participate.

### Data management and analysis

Data was collected using google.docs where questionnaire responses were converted to an excel spreadsheet, the learning environment created during hybrid learning was compared to previously collected, unpublished similar data for the learning environment created for a distance learning paediatric dentistry course, taught at the same university during the academic year 2019 /2020 (*n* = 166). The learning environment created to deliver paediatric dentistry hybrid course was correlated to students’ satisfaction data collected using SS.

Categorical data were presented as frequency and percentage values. Numerical data were represented as mean and standard deviation (SD) values. They were tested for normality using Shapiro-Wilk test. They had non-parametric distribution, so they were compared using Mann-Whitney U test. Spearman’s rank-order correlation coefficient was used to correlate students’ satisfaction and different DELES constructs. Multiple regression was used to predict satisfaction from different constructs. There was linearity as assessed by partial regression plots and a plot of studentized residuals against the predicted values. There was independence of residuals, as assessed by a Durbin-Watson statistic of 1.884. There was homoscedasticity, as assessed by visual inspection of a plot of studentized residuals versus unstandardized predicted values. There was no evidence of multicollinearity, as assessed by tolerance values greater than 0.1. There were no studentized deleted residuals greater than ±3 standard deviations, no leverage values greater than 0.2, and values for Cook’s distance above 1. The assumption of normality was met, as assessed by a Q-Q Plot. The significance level was set at *p* < 0.05 within all tests. Statistical analysis was performed with R statistical analysis software version 4.1.0 for Windows.

### Ethical considerations

**Fig. 1:**
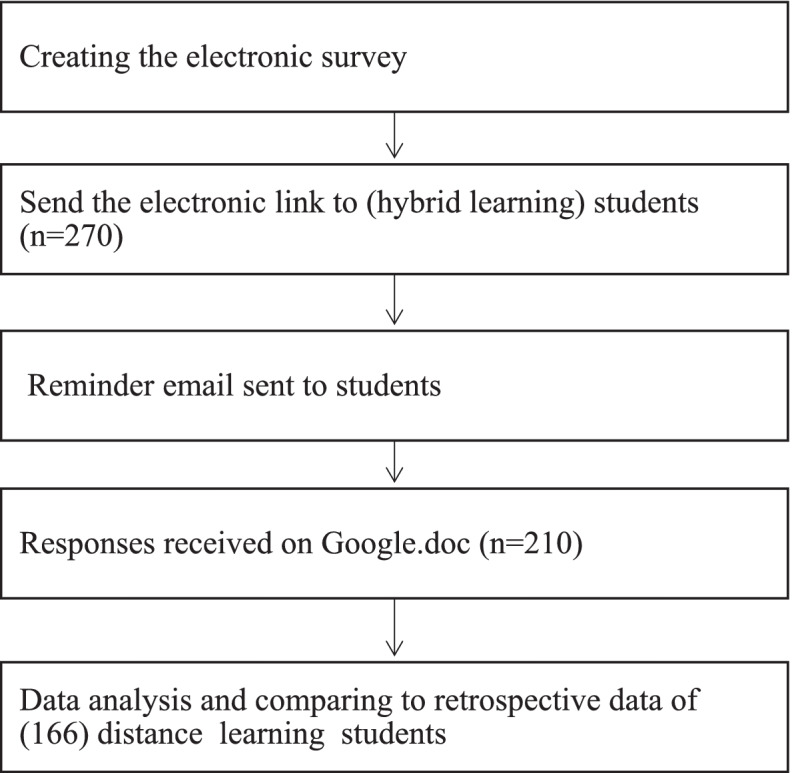
Study flow chart

This study has been registered on clinicaltrials.gov with an identifier: NCT04401371. The research was carried out in accordance with the Helsinki Declaration. Ethical approval was sought and approved from ethics committee, Faculty of Dentistry, Cairo University (19420), the study flowchart is presented in Fig. 1.

## Results

A total of 376 students took part in the questionnaire, their age ranges from 21 to 26 years (mean = 22.8). About 281(74.73%) are females and 95 (25.27%) of them are males. One hundred sixty-six out of the three hundred seventy-six students (44.14%) had distance learning, and 210 out of the 376 students (55.85%) had hybrid learning. The response rate among students who received distance learning was 46.8% (166 out 354), while among students who received hybrid learning it was 77.7% (210 out of 270). Descriptive statistics for demographic data were presented in Table 1.

**Table 1:** Descriptive statistics for demographic data

Respondents	*n* = 376	
Age	Mean = 22.8	Range = 21-26
**Gender**		
**Female**	*n* = 281	74.73%
**Male**	*n* = 95	25.27%
**Learning method**		
**Distance**	*n* = 166	44.14%
**Hybrid**	*n* = 210	55.85%

**Fig. 2:**
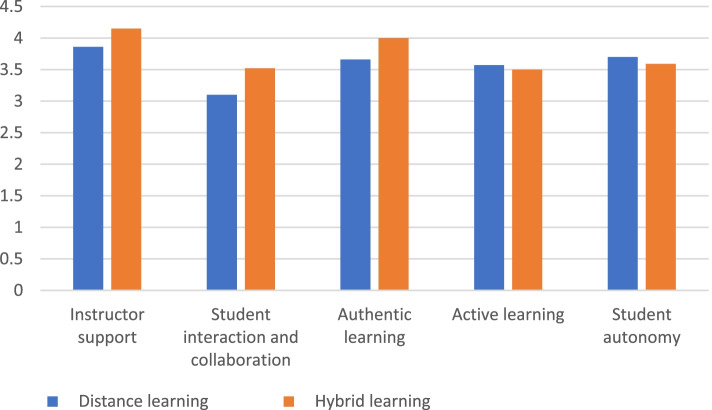
Bar chart showing average distance and hybrid education learning environment score

Hybrid learning had significantly higher DELES scores than distance learning regarding “Instructor support”, “Student interaction and collaboration” and “Authentic learning”. Intergroup comparisons and average values for DELES scores were presented in Table 2 and Fig. 2 respectively.

**Table 2:** Intergroup comparison for the educational learning environment score

Scale	(Mean ± SD)		Effect size (r)^	***p***-value
	***Distance learning***	***Hybrid learning***		
**Instructor support**	3.86 ± 0.94	4.15 ± 0.85	**0.156**	0.003*
**Student interaction and collaboration**	3.10 ± 1.17	3.52 ± 1.11	**0.182**	< 0.001*
**Authentic learning**	3.66 ± 1.13	4.00 ± 0.95	**0.148**	0.004*
**Active learning**	3.57 ± 1.01	3.50 ± 0.92	**0.093**	**0.455**
**Student autonomy**	3.70 ± 1.11	3.59 ± 1.03	**0.061**	**0.238**

^ *r* < 0.3 is considered small effect

*significant (*p* < 0.05)

Results of the correlation between students’ satisfaction and different constructs of DELES for the hybrid learning group were presented in Table 3. There was a statistically significant weak positive correlation between satisfaction and different constructs (*p* < 0.001). Correlation coefficient values ranged from (0.390) for “Student autonomy” to (0.250) for “Active learning”.

**Table 3:** Correlation between students’ satisfaction and different DELES constructs

Scale	Students’ satisfaction	
	***r***_***s***_	***p-value***
**Instructor support**	0.340	< 0.001*
**Student interaction and collaboration**	0.280	< 0.001*
**Authentic learning**	0.330	< 0.001*
**Active learning**	0.250	< 0.001*
**Student autonomy**	0.390	< 0.001*

*r*_*s*_ Spearman’s rank order correlation coefficient

^*^significant (*p* < 0.05)

Results of multiple regression analysis to predict students’ satisfaction from different DELES constructs were presented in Table 4. The overall model was statistically significant (*p* < 0.001) and accounted for (22.8%) of the variance in students’ satisfaction. Only “Instructor support” (*p* = 0.001) and “Student autonomy” (*p* < 0.001) had a significant effect on satisfaction.

**Table 4:** Regression analysis

	Students’ satisfaction			
	***Unstandardized coefficients***	***SE***	***Standardized coefficients***	***p-value***
**Intercept**	0.491	0.432		
**Instructor support**	0.385	0.119	0.26	0.001*
**Student interaction and collaboration**	0.077	0.087	0.07	**0.373**
**Authentic learning**	0.054	0.116	0.04	**0.641**
**Active learning**	−0.060	0.107	−0.04	**0.573**
**Student autonomy**	0.341	0.091	0.28	< 0.001*

*SE* Standard Error

^*^significant (*p* < 0.05)

## Discussion

The targeted population in our study included all senior dental students in Cairo University at the given academic year; a census study is free from sampling errors and will result in a more generalized insight [13].

DELES has a strong factorial validity, and high level of reliability, with Cronbach’s alpha coefficient ranging from 0.75 to 0.94. In general, this is not enough to consider DELES to have the best psychometric properties for different contexts. However, DELES was created to assess the learning environments in education programs and to correlate the nature of the distance education learning environment to students’ acceptance and enjoyment of their subjects, which is very close to the context of our study [11, 14, 15].

DELES has originally six constructs building up a 34-question survey; in the current study, the “personal relevance” construct was not used as we found that students had no chance to test their knowledge in relevance to real-life activities.

Students in the Faculty of Dentistry, Cairo University study in English and can easily read and comprehend English, that is why the English versions of DELES and SS were used.

Data reliability and security have been demonstrated in electronic versions of questionnaires [16, 17]. Some platforms, on the other hand, are costly. To address these issues, we adapted the Google Docs format to develop a useful computerized questionnaire system. It is simple, efficient, and the entry form can be designed to accommodate any type of questionnaire response.

Students’ responses showed statistically significant (*p* < 0.005) higher results for “Instructor support”, “student interaction and collaboration” and “authentic learning” scales for hybrid than for distance learning. The “active learning” and “student autonomy” scales showed close, non-statistically significant results (slightly higher for distance learning). These results came in agreement with Asiry [9], study where most students preferred a combination of traditional teaching methods and online learning. McCutcheon et al. [18], found that hybrid learning provides added educational value over online learning in an undergraduate nursing program. A randomized controlled trial by Moon et al. [19], found that a hybrid learning program succeeded in improving nursing students’ cardiopulmonary resuscitation knowledge.

Regarding the “instructor support” scale, there were significantly higher scores for hybrid learning versus distance learning. Students feel that they are getting more help, guidance, and feedback through face-to-face student-instructor interaction in hybrid learning. This comes in agreement with Lodge et al. [20], who believed that lack of face-to-face human interaction in pure distant education may lead to a negative effect on instructor-student interaction, which is a key factor for implementing instructor support.

“Student interaction and collaboration” scores were also higher for hybrid learning than distance learning agreeing with Furnborough [21], who reported that in online classes spontaneous interaction between students is not as simple as in conventional classes. Hybrid learning methodology usually encourages group tasks in the form of group assignments and discussions, which can facilitate group cohesion and student collaboration.

“Authentic learning” had significantly higher scores for hybrid learning than for distance learning. This was disagreeing with a previous interesting study by Kartoğlu [22], who used the shift to online public health course to increase the time available for “on-site mock for (Good Clinical Practices) inspection” from 6-h practical session to 24-week online interactive simulation. Authentic learning aims to engage students with real-life activities to practice what they are learning, this occurs daily in medical and dental education, with the simplest form being problem-based education, where real-life clinical situations and challenges are used to allow students to apply and test their theoretical knowledge [23].

For the correlation between students’ satisfaction and the created environment for the hybrid learning group, there was a statistically significant (*p* < 0.001) weak positive correlation between SS and all scales of DELES. This came in agreement with Qutieshat et al [24], study which compared hybrid learning and traditional learning methods for fourth-year dental students, students accepted hybrid learning well and ranked it high for satisfaction and usefulness.

Moreover, results of multiple regression analysis (Table 4) showed that the learning environment is a predictor for satisfaction (22.8%) in a hybrid course, specifying that “Instructor support” (*p* = 0.001) and “Student autonomy” (*p* < 0.001) had a significant association with satisfaction. These findings support previous data by Venkatesh et al. [25], who also concluded that the learning environment is a predictor of students’ satisfaction; also, agreeing with Gunawardena and Zittle [26] who found that “student perception of having equal opportunity to participate” can be considered as a predictor for students’ satisfaction.

When instructors support autonomy, students have more opportunities to take control because autonomy fosters greater enthusiasm, interest, and a desire for challenge, allowing students to develop self-determined motivation and meet their basic psychological needs [27]. Additionally, students are more likely to rate courses as satisfactory in the presence of good communication with instructors, if their courses are well organized, and if their instructors expressed interest in their learning and respected them [28].

It is worth mentioning that the study has some limitations; the non-response bias where some students included in the sample didn’t respond to the questionnaire and their response could have affected the results, and the study took place during the COVID-19 pandemic where decisions to shift to different educational approaches was spontaneous rather than well planned.

This study gives an insight into students’ opinions regarding the learning environment created to deliver a paediatric dentistry course. The results of the current study suggest that a hybrid learning environment is more accepted and satisfying to students than distance learning. With the help of previous similar work and future work to come, the process of implementing hybrid learning for undergraduate or continuing education could be done in the field of dental education in other than emergency conditions, which would be one step forward in the way of solving the problem of deficient oral health care workers in some countries. One should stress that a lot of work and studies should be done to be able to implement such programs without jeopardizing the educational quality.

Future work is needed to build on the results of the current study, more studies on different courses, studies involving 100% on-campus learning in comparison to hybrid learning, specific studies to suggest which topics are more suitable for online learning and which should be taught on-campus, studies testing different available on-line learning applications and tools, and what is the added value for using any of these tools will be beneficial as well. Instructors’ preferences and satisfaction should be studied for all the aforementioned points.

## Conclusions

The results of this study support the superiority of a hybrid learning environment when compared to the distance learning environment created to teach paediatric dentistry course, it also shows that satisfaction is correlated and can be predicted by the created learning environment.

## Supplementary Information

**Additional file 1.**

## Data Availability

The datasets generated and analyzed during the current study are not publicly available due to ethical and privacy considerations but are available from the corresponding author on reasonable request.

## Abbreviations

DELES: Distance Educational Learning Environment Survey
SS: Satisfaction Scale
