# Supplementary material for: Hybrid versus distance learning environment for a paediatric dentistry course and its influence on students’ satisfaction: a cross-sectional study
Source: BMC Med Educ. 2022 May 5;22:343. doi: 10.1186/s12909-022-03417-4 (PMC9069422; doi:10.1186/s12909-022-03417-4)
Supplement: Supplementary file 1 — Additional file 1. [file 12909_2022_3417_MOESM1_ESM.docx]

DISTANCE EDUCATION LEARNING ENVIRONMENTS SURVEY (DELES) AND SATISFACTION SCALE (SS)

Instructor support

In this class *. . .*

1. If I have an inquiry, the instructor finds time to respond.

Always Often Sometimes Seldom Never.

1. The instructor helps me identify problem areas in my study.

Always Often Sometimes Seldom Never.

1. The instructor responds promptly to my questions.

Always Often Sometimes Seldom Never.

1. The instructor gives me valuable feedback on my assignments.

Always Often Sometimes Seldom Never.

1. The instructor adequately addresses my questions.

Always Often Sometimes Seldom Never.

6. The instructor encourages my participation.

Always Often Sometimes Seldom Never.

7. It is easy to contact the instructor.

Always Often Sometimes Seldom Never.

8. The instructor provides me with positive and negative feedback on my work.

Always Often Sometimes Seldom Never.

Student interaction and collaboration

In this class *. . .*

9. I work with others.

Always Often Sometimes Seldom Never.

1. I relate my work to others’ work.

Always Often Sometimes Seldom Never.

1. I share information with other students.

Always Often Sometimes Seldom Never.

1. I discuss my ideas with other students.

Always Often Sometimes Seldom Never.

1. I collaborate with other students in the class.

Always Often Sometimes Seldom Never.

14. Group work is a part of my activities.

Always Often Sometimes Seldom Never.

Authentic learning

In this class *. . .*

1. I study real cases related to the class.

Always Often Sometimes Seldom Never.

1. I use real facts in class activities.

Always Often Sometimes Seldom Never.

1. I work on assignments that deal with real-world information.

Always Often Sometimes Seldom Never.

18. I work with real examples.

Always Often Sometimes Seldom Never.

1. I enter the real world of the topic of study.

Always Often Sometimes Seldom Never.

Active learning

In this class *. . .*

1. I explore my own strategies for learning.

Always Often Sometimes Seldom Never.

21. I seek my own answers.

Always Often Sometimes Seldom Never.

22. I solve my own problems.

Always Often Sometimes Seldom Never.

Student autonomy

In this class *. . .*

23. I make decisions about my learning.

Always Often Sometimes Seldom Never.

1. I work during times that I find convenient.

Always Often Sometimes Seldom Never.

1. I am in control of my learning.

Always Often Sometimes Seldom Never.

1. I play an important role in my learning.

Always Often Sometimes Seldom Never.

1. I approach learning in my own way.

Always Often Sometimes Seldom Never.

Satisfaction scale

1. Distance education is stimulating

Strongly agree Agree Neither agree nor disagree Disagree Strongly disagree.

1. I prefer distance education

Strongly agree Agree Neither agree nor disagree Disagree Strongly disagree.

1. Distance education is exciting

Strongly agree Agree Neither agree nor disagree Disagree Strongly disagree.

1. Distance education is worth my time

Strongly agree Agree Neither agree nor disagree Disagree Strongly disagree.

1. I enjoy studying by distance

Strongly agree Agree Neither agree nor disagree Disagree Strongly disagree.

1. I look forward to learning by distance

Strongly agree Agree Neither agree nor disagree Disagree Strongly disagree.

1. I would enjoy my education more if all my classes were by distance

Strongly agree Agree Neither agree nor disagree Disagree Strongly disagree.

1. I am satisfied with this class

Strongly agree Agree Neither agree nor disagree Disagree Strongly disagree.

Reference

Walker, S. L., & Fraser, B. J. (2005). Development and validation of an instrument for assessing distance education learning environments in higher education: The Distance Education Learning Environments Survey (DELES). *Learning Environments Research, 8*, 289–308.

Short J. A., Williams, E., & Christie, B. The Social Psychology of Telecommunications. London, England; Wiley; 1976.
